# Rate of Corneal Collagen Crosslinking Redo in Private Practice: Risk Factors and Safety

**DOI:** 10.1155/2015/690961

**Published:** 2015-03-19

**Authors:** Joelle Antoun, Elise Slim, Rami el Hachem, Elias Chelala, Elyse Jabbour, Georges Cherfan, Elias F. Jarade

**Affiliations:** ^1^Saint Joseph University Hospital, Faculty of Medicine, P.O. Box 166830, Beirut, Lebanon; ^2^Beirut Eye Specialist Hospital, Al-Mathaf Square, P.O. Box 116-5311, Beirut, Lebanon; ^3^Mediclinic, Dubai Mall, Dubai, UAE

## Abstract

*Objective*. To report the rate of progression of keratectasia after primary crosslinking (CXL) and evaluate the safety and efficiency of CXL redo. *Materials and Methods*. We conducted a retrospective analysis of the patients who underwent CXL between 2010 and 2013 at the Beirut Eye Specialist Hospital, Lebanon. Progression of keratectasia was based on the presence of an increase in maximum keratometry of 1.00 D, a change in the map difference between two consecutive topographies of 1.00 D, a deterioration of visual acuity, or any change in the refraction. Primary and redo CXL were done using the same protocol. *Results*. Among the 221 eyes of 130 patients who underwent CXL, 7 eyes (3.17%) of five patients met the criteria of progression. All patients reported a history of allergic conjunctivitis and eye rubbing and progressed within 9 to 48 months. No complications were noted and all patients were stable 1 year after CXL redo. *Conclusion*. Allergic conjunctivitis and eye rubbing were the only risk factors associated with keratoconus progression after CXL. A close followup is thus mandatory, even years after the procedure. CXL redo seems to be a safe and efficient technique to halt the progression after a primary CXL.

## 1. Introduction

Keratoconus (KC) is a noninflammatory corneal disease characterized by corneal deformation and thinning caused by structural changes in the corneal collagen, inducing irregular astigmatism, myopia, and protrusion, which leads to mild to marked impairment in vision quality [1]. Corneal ectasia is one of the most devastating complications after laser-assisted in situ keratomileusis (LASIK). The disease is characterized by a progressive thinning and steepening of the central and inferior portions of the cornea, inducing a loss of uncorrected visual acuity, best-corrected visual acuity, and topographic evidence of asymmetric inferior corneal steepening [2–5].

Corneal collagen crosslinking (CXL) was introduced in 2003 by Wollensak et al. to halt the progression of keratectasia [6–8]. During CXL, riboflavin interacts with ultraviolet-A (UV-A) light to cause crosslinking of protein fibrils followed by formation of interchain disulfide bonds, thus arresting the progression of keratoconus by increasing the biomechanical stability of the cornea [7, 8]. In the meantime, CXL has become an increasingly well-accepted low invasive intervention with high success and low complication rates [9–11]. Long-term stabilization and improvement after CXL have been reported in many prospective studies [11–13]. However, failure and progression of keratectasia after CXL have been reported. Recently, Kymionis et al. [14] reported that topographic keratoconus progression might occur several years after CXL, despite stability for a long-term period. While Greenstein and Hersh showed that no preoperative characteristics were predictive of CXL failure for keratectasia [15], other studies attributed progression after primary CXL to specific risk factors and patient characteristics. Koller et al. reported an 8% CXL failure rate one year after CXL for keratoconus, with preoperative maximum *K* value of more than 58.0 D as a risk factor for progression [16]. In addition, due to the few cases of progression after CXL, there is no consensus about the definition of progression of keratectasia after CXL. The number of cases of keratoconus progression after original CXL procedure is expected to increase with time with no clear consensus about the best treatment modality of those cases.

To our knowledge, no previous study evaluated the safety and efficiency of CXL redo. We hence report the rate of progression and the risk factors after CXL in our private practice and evaluate the technique, safety, and efficiency of CXL redo after primary CXL.

## 2. Materials and Methods

### 2.1. Setting

We conducted a retrospective analysis of the patients with progressive keratectasia who underwent CXL between March 2010 and March 2013 at the Beirut Eye Specialist Hospital Beirut, Lebanon. Diagnosis of keratoconus was based on a combination of computed slit-scanning videokeratography of the anterior and posterior corneal surfaces, keratometric readings, and corneal pachymetry. Keratoconus was classified into four stages based on corneal power, astigmatism, corneal transparency, and corneal thickness, according to the classification of Amsler-Krumeich [17].

### 2.2. Participants

All patients included in this study had a history of progressive keratectasia either from keratoconus or from corneal ectasia after LASIK in one or both eyes and underwent a primary CXL in order to stabilize the disease.

All patients included in this study had a central corneal thickness >400 *μ*m. Central corneal thickness (CCT) and thinnest corneal location were measured using Pentacam topography with epithelium on prior to the procedure. Because of the potential deswelling effect in the corneal stroma of the dextran in riboflavin solution [18], the CCT and thinnest corneal location were measured using ultrasound pachymeter all through the period of riboflavin application during the entire period of CXL procedure. Therefore, after removing the epithelium, CCT and thinnest corneal location were measured although the period of riboflavin application and hypoosmolar riboflavin was additionally instilled every 20 seconds for 5 minutes and repeated up to 2 times until adequate minimal corneal thickness of more than 400 *μ*m was reached.

Exclusion criteria for primary and redo CXL were preoperative corneal opacities, ocular pathology other than keratectasia, especially the cornea guttata or other endothelial irregularities, age younger than 18 years, actual or intended pregnancy, not available for follow-up examinations for 1 year, and connective tissue disease.

### 2.3. Data Collection

Progressive keratectasia was suspected by an increase in maximum *K* readings in several consecutive recordings in the last 6 months with or without progressive corneal thinning as well as deterioration in visual acuity and manifest refraction. We evaluated the progression of KC after CXL based on the presence of 2 or more of the following criteria: increase (≥1 D) in *K* value (*k*1, *K*2, or *K* max), a change in the map difference between two consecutive topographies of ≥1 D, a deterioration of VA defined as a drop of one or more lines, or any change in the refraction as a change of 0.5 D.

Based on our observation, the cornea may endure major topographic changes in the first 6 months after CXL with significant changes in manifest refraction and visual acuity. We set a baseline corneal topography 6 month after CXL treatment and we noticed no further clinically significant changes are happening then after apart from a nonsignificant minor flattening of *k*-reading over time with no further steepening happening 6 months after CXL treatment in stable keratoconus. Any steepening in *k*-reading that happens 6 months after primary CXL is considered as sign of keratoconus progression, mainly when it was associated with one of the aforementioned criteria of keratoconus progression. Therefore, the effect of CXL is considered only 6 months after primary CXL and corneal stability is judged after that.

All patients undergoing CXL were followed up closely in the postoperative period at day one, one week, one month, 3 months, sixth months, and one year and then half yearly after. Corneal topography was repeated at each visit starting 3 months after CXL and then 6 months after CXL corneal topography is considered as the baseline topography after CXL based on which the progression of KC is considered. Thus, all patients are evaluated at 6 months after CXL by a complete ophthalmic workup including assessment of uncorrected distance visual acuity (UDVA), corrected distance visual acuity (CDVA), manifest and cycloplegic refractions, and anterior and posterior segment evaluation with dilated fundus examination, as well as an anterior/posterior topography. Corneal topography (Pentacam 70700, Oculus, Germany) was conducted with undilated pupils under scotopic conditions by a single experienced technician. Baseline strategy for the treatment of recurrent allergic episodes was based on topical mild steroids (Fluorometholone 0.1%) to be used four times a day for 10 to 14 days as needed. In case of severe exacerbations, topical antihistamine and topical cyclosporine 0.1% were added to the regimen.

### 2.4. Surgical Procedure

Primary and redo corneal collagen crosslinking (CXL) were done using the same protocol. All surgeries were performed by the same surgeon (EJ). The eye to be treated was anesthetized by applying proparacaine hydrochloride 0.5% drops on three occasions spaced by five minutes. After positioning the patient under the operating microscope, a lid speculum was inserted and the central 9 mm corneal epithelium was removed with a blunt spatula. A mixed riboflavin 0.1% dextran solution (Collagex, isotonic 0.1%, Lightmed USA Inc.) was instilled every 2 minutes until the riboflavin penetrated the cornea, that is, after approximately 30 minutes. The ultraviolet (UV) lamp (UV-X illumination system, version 1000; IROC AG, Zurich, Switzerland) was then focused on the apex of the cornea at a distance of 5 cm for a total of 30 minutes, providing a radiant energy of 3.0 ± 0.3 mW/cm^2^. The required irradiance of 3.0 mW/cm^2^ was calibrated prior to each treatment using a UVA meter (LaserMate-Q; LASER 2000, Wessling, Germany). During UVA administration, riboflavin drops were applied to the cornea every 2 minutes. Thinnest and central pachymetry were continuously monitored to ensure that none of the two parameters dropped below 400 *μ*m. After treatment, the eye surface was washed with balanced salt solution and two drops of gatifloxacin 0.3% were instilled, followed by the placement of a bandage soft contact lens. Postoperatively, patients received acetaminophen 500 mg twice daily for 3 days, one drop of gatifloxacin 0.3% six times daily for 7 days along with one drop of tobramycin-dexamethasone 0.1% four times daily for 10 days, followed by one drop of Loteprednol 0.5% 5 times daily, slowly tapered over 5 weeks. The bandage soft contact lens was removed on postoperative day 4, and the eye examined by slit-lamp microscopy to confirm complete corneal epithelialization.

Complications after CXL redo such as significant stromal haze, sterile corneal infiltrates, recurrent erosion syndrome, corneal edema, Desçemets membrane folds, corneal melting and perforation were noted if present. Stability after CXL redo was also assessed.

## 3. Results

### 3.1. Primary CXL

Two hundred twenty-one eyes of 130 patients underwent a corneal collagen CXL for progressive keratoconus or post-LASIK ectasia in our department between March 2010 and March 2013. The demographic and topographic data of the initial 221 eyes are mentioned in Table 1.

### 3.2. CXL Redo

Although the majority of the eyes remained stable after primary CXL (according to the aforementioned criteria), seven eyes (3.17%) of five patients met the criteria of progression and necessitated a CXL redo. Patients characteristics are summarized in Table 2. Mean age was 26 (one patient was 19 years old and 4 patients were aged between 26 and 30 years), with male/female ratio of 3/2. All patients who progressed reported a history of allergic conjunctivitis and eye rubbing. Their preoperative maximum *K* value was > 58.0 D in 3 eyes and <58 D in 4 eyes (mean of 58.6 D). Two eyes (of one patient) had CXL alone, 4 eyes had CXL subsequent to ICRS implantation, and one eye had simultaneous CXL with PRK. Four eyes had a stage 2 keratoconus, 2 eyes had a stage 4 keratoconus, and one eye had a post-LASIK ectasia.

**Figure 1:**
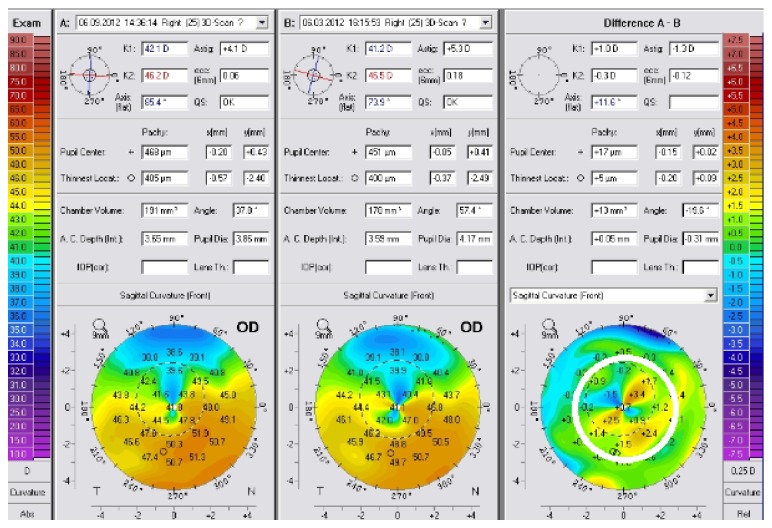
A map difference showing progression after primary corneal collagen crosslinking. B represents corneal topography 6 months after primary CXL, and A shows corneal topography 12 months after primary CXL. The map difference (difference A-B) shows the progression after initial CXL with +2.5 and +3.4 D of difference between successive topographies (white circle).

Progression of KC was noticed more than one year (14 to 48 months) after the original CXL in 6 eyes of four patients (2 males and 2 females, one patient was 19 years old, and 3 were 26–28 years old) and one eye (30-year-old male) was diagnosed with KC progression 9 months after the original CXL. The mean time of KC progression after original CXL was 29.14 months. Progression was noted by all the patients after a decrease in CDVA and was evidenced by progression in corneal topography (Figure 1). Progression was simultaneously noted in both eyes in all patients who had bilateral disease evolution.

No major complications after CXL do and redo such as significant stromal haze, sterile corneal infiltrates, recurrent erosion syndrome, corneal edema, Desçemets membrane folds, corneal melting and perforation were noted in any patient. At one year after CXL redo, all patients remained stable by either UDVA, CDVA, or topographic readings. The characteristics of the 5 patients are summarized in Tables 2, 3, and 4.

## 4. Discussion

Despite the proven effect of CXL in halting the progression of KC and corneal ectasia with stabilization in the majority of cases [7, 19, 20], KC progression still can happen after primary CXL treatment [14, 16]. In most of the studies, the reported failure rate varied from 0% [6, 21] to 16.5% [22]. The time of progression after CXL was reported to be as early as few months [16] to 5 years after CXL [14]. The most adopted definition of KC progression after CXL in most of the reports in the literature was an increase in the maximum keratometry readings of >1.00 D over the 6 months after CXL value [14, 16]. In our study, 2 eyes (eye 1 and eye 4) presented with evidence of progression based on worsening of their visual acuity despite a progression of their *k* readings of less than 1.00 D (0.9 in eye 1 and 0.68 in eye 2). Thus, a change in the map difference between two consecutive topographies of 1.00 D (or maybe less), a deterioration of visual acuity (excluding other possible non-cornea-related reasons for deterioration), or any change in the refraction must be taken into account when evaluating the stability after CXL. Any of these indicators are considered as progression and necessitate a redo of CXL. The failure of CXL as a continued progression of keratoconus during the first year postoperatively has been reported in several studies [16, 23, 24]. The failure rate has been reported to be around 7 to 9%. Koller et al. reported a failure rate of 7.6% during the first postoperative year [16]. Hersh et al. and Sloot et al. [23, 24] reported a failure rate of 9.8% and 9%, respectively, while Baenninger et al. reported a failure rate of 16.5% in patients aged <35 years [22]. In our study, we found that the failure rate is 3.17% which is significantly less than the rates in the previous reports. Although our CXL technique is the same technique described in the aforementioned reports, the lower failure rate at our practice can be attributed to any of the following factors. First, this chart review was performed in our private clinic, and the lower failure rate might be due mostly to the fact that unhappy progressive keratoconus patients might be lost to followup. Second, many of the patients at our private clinic had CXL associated refractive surgeries (32%), such as PRK (9%) or ICRS (23%). Few reports imply not only the safety of the latter procedures, but also their possibility to add up to CXL's collagen stabilization [25–28]. Such procedures might have reduced our failure rate. Third, we considered 6 months after CXL as baseline data and KC progression was judged based on the corneal topography performed at 6 months after initial CXL. Therefore, we might have reduced the selection error due to the keratometric fluctuation during the first 6 months after CXL, which may have contributed to the lower rate of KC progression in our study. In fact, some studies reported that the initial fluctuation and maybe worsening of keratometric readings are observed in the first months following CXL [16]. This change may be due to transient haze, corneal edema, and remodeling [29, 30]. Accordingly, we evaluated significant changes in keratometric values for assessment of CXL efficacy only 6 months after CXL.

Risk factors associated with progression after primary CXL remain unclear. In our practice, a history of allergic conjunctivitis with eye rubbing was found to be a common risk factor to all patients in the progression group. However, because of the small number of patients with keratoconus progression, we could not conduct a multifactorial analysis to determine other risk factors. Further prospective studies with multifactorial analysis are thus necessary to determine other risk factors associated with progression of keratoconus after a primary CXL. Similarly, Raiskup-Wolf et al. reported progression in 2 patients with neurodermatitis, a condition in which constant skin and ocular rubbing is present [13]. In fact, the relationship between eye rubbing and keratoconus has been studied in previous reports [30, 31]. Eye rubbing leads to biomechanical and biochemical alterations [32]. It injures the epithelium and leads to cytokine and metalloproteins release [32]. Stromal thinning occurs and this contributes to the keratoconus disease progression [32]. In our case, we think that the eye rubbing and the mechanical trauma it caused played an important role in the recurrence of the disease [31–33]. Other postulated factors for progression such as female sex and elevated maximal keratometry were not predominant factors in our study. In the study of Koller et al. there were differences between the failure subgroup with the total group in sex, where females had significantly more failure rates than males (females 62.5% versus 38.8% in males; *P* = 0.048), and preoperative maximum *K* reading of less than 58.00 D was found to reduce the failure rate to 3% [34]. In our cases, the gender was not a risk factor for progression (3 males, 2 females), nor the *K* max (*K* max >58 D in 2 eyes and <58 D in 3 eyes). Finally, one out of the five patients in our study with CXL failure in our study had a post-LASIK ectasia. Post-LASIK ectasia might have a higher rate of failure. Hersh et al. reported a reduced effect of CXL in cases of post-LASIK ectasia compared with keratoconus [23]. It was postulated that the reduced effect could be due to the influence of the flap, which may impede the diffusion of riboflavin or change the behavior of the anterior stroma to the crosslinking process [23]. Finally, the small group of failure makes multivariate analysis nonconclusive. In our study, 5 out of 7 eyes had associated surgeries, 4 eyes had CXL subsequent to ICRS implantation, and one eye had simultaneous CXL with PRK. These two types of associated surgeries were not found to increase failure rate in literature reviews and are considered safe in combination with CXL in keratectasia [11, 35, 36].

The majority of the studies report the failure of CXL during the first year postoperatively [16, 22, 24]. In a recent paper published by Kymionis et al., a topographic examination revealed an increase in the keratometric values indicating keratoconus progression 4 and 5 years after CXL, despite stability for a long-term period. In our series, four patients presented with a progression time after crosslinking ranging from 14 months to 48 months. To our knowledge, this is the second case series reported in the literature, in which patients with stability after CXL for a long-term period showed topographic recrudescence. The exact pathophysiology of keratoconus progression after years of stability following CXL is not known but could be related to the new collagen laydown. Richoz et al. evoked the role of corneal stromal regeneration and rejuvenation as a possible explanation in the recurrence of the disease [8]. Also, in our study we found that the patient's age is not predictive of failure; the younger patient in our series (19 years old) had CXL failure at 38 months postop, while patients around 30 years old had failure at different times 9 to 48 months.

In the literature, the safety, efficiency, and the technique of CXL redo were not previously evaluated. To our knowledge, this is the first report to assess long-term safety and efficacy of CXL redo. We performed the primary and redo corneal collagen CXL using the same classical protocol, and the 7 eyes we treated with CXL redo were stable 1 year after the second CXL. No complications after CXL redo such as significant stromal haze, sterile corneal infiltrates, recurrent erosion syndrome, corneal edema, Desçemets membrane folds, corneal melting and perforation were noted in any of the patients we treated with CXL redo. However, we did not perform an endothelial cell count preoperatively and after CXL and the effect of CXL redo on the endothelial health was not evaluated which constitutes a limitation to our study.

Recently, Kanellopoulos and Asimellis introduced a novel, noninvasive, quantitative technique utilizing anterior segment OCT images to quantitatively assess the depth and cross-sectional area of CXL in the corneal stroma. Despite the usefulness of the aforementioned method, OCT was not performed systematically in all patients who underwent CXL treatment in our study; therefore, the value of OCT in determining the depth and effectiveness of CXL treatment was not studied in our group, and this is considered as a limitation factor of our study [37].

## 5. Conclusion

In conclusion, according to our understanding of keratocytes turnover in the cornea, the effect of CXL may be transient and progression of KC after primary CXL may happen. Thus, a close followup is mandatory in patients after CXL, even after a stability of years after the procedure and CXL redo procedures for those cases who progressed seems to be a safe and efficient technique to halt the progression of keratoconus or post-LASIK corneal ectasia after a failed primary CXL.

## Figures and Tables

**Table 1:** Baseline patient characteristics of 221 eyes of 130 patients who underwent CXL between March 2010 and March 2013 at our private clinic. PRK: photorefractive keratectomy; ICRS: intrastromal corneal ring segments; *n*%: number (percentage).

Characteristics	Value
Gender	
Male (*n*%)	68 (52%)
Female (*n*%)	62 (48%)
Age, years	
16–30	95 (73.1%)
30–50	35 (26.9%)
Stage of KC (Amsler-Krumeich)	
Stage 1 (*n*%)	96 (43%)
Stage 2 (*n*%)	100 (45%)
Stage 3 (*n*%)	25 (12%)

Keratometry, diopters	
Flattest meridian	
40–44	132 (60%)
44–46	89 (40%)
Steepest meridian	
45–58	122 (55%)
58–68	99 (45%)
Maximal keratometry	
48–58	103 (47%)
58–68	118 (53%)

Pachymetry, microns	
400–450	156 (70%)
450–580	65 (30%)
Associated surgeries *n*%	70 (32%)
ICRS *n*%	50 (23%)
PRK *n*%	20 (9%)

**Table 2:** Patient characteristics: all eyes had allergic conjunctivitis. ∗: missing data. CCT: central corneal thickness; PRK: photorefractive keratectomy; ICRS: intrastromal corneal ring segments; LASIK: laser-assisted in situ keratomileusis.

Eye	Age	Gender	Diagnosis	Stage of Kc.	Baseline CCT	**Allergic conjunctivitis**	Associated surgeries	Time (months) since first CXL	Slit lamp evaluation (first visit)
Eye 1	27	F	Keratoconus	Stage 2	489	Yes	PRK	14	No haze papillae
Eye 2	19	F	Keratoconus	Stage 2	523	Yes	ICRS	38	No haze papillae
Eye 3	30	M	Post-LASIK ectasia		420	Yes	ICRS	9	No haze papillae
Eye 4	26	M	Keratoconus	Stage 4	414	Yes	None	23	Striae papillae
Eye 5	26	M	Keratoconus	Stage 4	418	Yes	None	24	Striae + haze papillae
Eye 6	28	M	Keratoconus	Stage 2	∗	Yes	ICRS	48	No haze papillae
Eye 7	28	M	Keratoconus	Stage 2	∗	Yes	ICRS	48	No haze papillae

**Table 3:** Progression of keratometric readings. *K* readings remained stable one year after CXL redo. ∗: missing data. #: difference.

Eye	Preoperatively			6 months after CXL			At diagnosis of progression			**Sign of progression **	One year after CXL redo		
	*K*1	*K*2	*k*max	*K*′1	*K*′2	*K*′max	*K*′′1	*K*′′2	*K*′′max		*K* ^∧^1	*K* ^∧^2	*K* ^∧^3
Eye 1	40.6	50.2	53.9	41.2	45.5	50.7	42.1	46.2	51.3	**#** **k**1**: 0.9 D,** **#** **K** **max 0.6 D** **↓CDVA**	42.00	45.8	51.00
Eye 2	45.2	48.9	51.3	42.93	45.45	∗	43.15	46.67	∗	**#** **k**2** 1.22 D **	43.00	46.87	∗
Eye 3	53.53	60.25	67.5	40.84	41.16	45.22	40.5	43.38	49.97	**#** **k**2**: 1.84 D** ** #** **k** **max 4.75 D **	40.00	43.25	49.80
Eye 4	51.6	55.1	57.1	52.7	54.38	∗	53.2	55.06	∗	**#** **k**1** 0.5 D #** **k** **max 0.68 D** **↓CDVA**	52.98	54.9	∗
Eye 5	54.5	56.8	63.2	48.3	51.4	∗	57.05	59.33	∗	**#** **k**1** 8.75 D** **#** **k**2** 7.92 D **	56.8	59.2	∗
Eye 6	∗	∗	∗	44.07	47.45	59.4	44.49	47.5	62.11	**#** **k** **max 2.71 D**	44.3	46.9	61.89
Eye 7	∗	∗	∗	47.69	50.05	59.51	47.74	49.71	61.7	**#** **k** **max 2.19 D **	47.8	49.59	61.2

**Table 4:** Change in error of refraction (EOR), uncorrected distance visual acuity (UDVA), corrected distance visual acuity (CDVA) from baseline to one year after CXL redo. UDVA and CDVA remained stable one year after CXL redo. ∗: missing data; S: sphere; C: cylinder; A: axis.

Eye	EOR													UDVA/**CDVA**			
	Baseline			6 mo CXL + associated surgeries			Refraction at progression									
	S	C	A	S′	C′	A′	S′′	C′′	A′′	Baseline		6 months after		Time of progression		1 year after CXL redo	
Eye 1	**−1.5**	**+4.25**	**170**	**−0.5**	**+1.00**	**20**	**−2.75**	**+2.75**	**175**	*20/100 *	**20/30**	*20/25 *	**20/20**	*20/100 *	**20/40**	*20/100 *	**20/40**
Eye 2	**−7.00**	**+3.50**	**150**	**−3.00**	**+1.75**	**170**	**−2.75**	**+2.75**	**10**	*20/200 *	**20/30**	*20/70 *	**20/40**	*20/80 *	**20/40**	*20/100 *	**20/40**
Eye 3	**−9.00**	**+3.50**	**20**	**−0.5**	**+0.75**	**170**	**−0.50**	**+3.00**	**135**	*20/400 *	**20/200**	*20/25 *	**20/20**	*20/50 *	**20/30**	*20/50 *	**20/25**
Eye 4	**−13.00**	**+2.00**	**75**	**−13.00**	**+1.75**	**70**	**−16.00**	**+1.5**	**70**	*CF *	**20/50**	*CF *	**20/30**	*CF *	**20/30**	*CF *	**20/30**
Eye 5	**−14.50**	**+0.75**	**90**	**−13.50**	**+1.75**	**70**	**−18.00**	**+2.5**	**55**	*CF *	**20/50**	*CF *	**20/30**	*CF *	**20/30**	*CF *	**20/40**
Eye 6	∗	∗	∗	**−4.00**	**+2.75**	**105**	**−5.00**	**+3.25**	**105**	∗	∗	*20/50 *	**20/25**	*20/70 *	**20/25**	*20/60 *	**20/30**
Eye 7	∗	∗	∗	**−1.75**	**+2.25**	**170**	**−3.50**	**+4.00**	**170**	∗	∗	*20/40 *	**20/30**	*20/100 *	**20/30**	*20/100 *	**20/25**
